# Chronic bacterial prostatitis: efficacy of short-lasting antibiotic therapy with prulifloxacin (Unidrox®) in association with saw palmetto extract, lactobacillus sporogens and arbutin (Lactorepens®)

**DOI:** 10.1186/1471-2490-14-53

**Published:** 2014-07-19

**Authors:** Gian Maria Busetto, Riccardo Giovannone, Matteo Ferro, Stefano Tricarico, Francesco Del Giudice, Deliu Victor Matei, Ottavio De Cobelli, Vincenzo Gentile, Ettore De Berardinis

**Affiliations:** 1 Department of Urology, Sapienza Rome University, Rome, Italy; 2 Department of Urology, European Oncology Institute, Milan, Italy; 3 Policlinico Umberto I, Sapienza Rome University, viale del Policlinico, 155, 00161 Rome, Italy

## Abstract

**Background:**

Bacterial prostatitis (BP) is a common condition accounting responsible for about 5-10% of all prostatitis cases; chronic bacterial prostatitis (CBP) classified as type II, are less common but is a condition that significantly hampers the quality of life, (QoL) because not only is it a physical condition but also a psychological distress. Commonly patients are treated with antibiotics alone, and in particular fluoroquinolones are suggested by the European Urology guidelines. This approach, although recommended, may not be enough. Thus, a multimodal approach to the prolonged antibiotic therapy may be helpful.

**Methods:**

210 patients affected by chronic bacterial prostatitis were enrolled in the study. All patients were positive to Meares-Stamey test and symptoms duration was > 3 months. The purpose of the study was to evaluate the efficacy of a long lasting therapy with a fluoroquinolone in association with a nutraceutical supplement (prulifloxacin 600 mg for 21 days and an association of Serenoa repens 320 mg, Lactobacillus Sporogens 200 mg, Arbutin 100 mg for 30 days). Patients were randomized in two groups (A and B) receiving respectively antibiotic alone and an association of antibiotic plus supplement.

**Results:**

Biological recurrence at 2 months in Group A was observed in 21 patients (27.6%) and in Group B in 6 patients (7.8%). Uropathogens found at the first follow-up were for the majority Gram – (E. coli and Enterobacter spp.). A statistically significant difference was found at the time of the follow-up between Group A and B in the NIH-CPSI questionnaire score, symptoms evidence and serum PSA.

**Conclusions:**

Broad band, short-lasting antibiotic therapy in association with a nutritional supplement (serenoa repens, lactobacillus sporogens and arbutin) show better control and recurrence rate on patients affected by chronic bacterial prostatitits in comparison with antibiotic treatment alone.

**Trial registration:**

NCT02130713

Date of trial Registration: 30/04/2014

## Background

Bacterial prostatitis (BP) is a common condition in which an infective origin is accepted, and accounts for about 5-10% of all prostatitis cases [1]. This is one of the most common reasons for younger men to consult with a urologist. Regarding duration of symptoms, we describe two main types: acute and chronic. In particular, when chronic, symptoms last more than 3 months and in agreement with the National Institute of Health (NIH) chronic bacterial prostatitis (CBP) is classified as type II. Even if in many cases the aetiology is known, in others there are only these symptoms: pelvic area pain and lower urinary tract symptoms (LUTS). Chronic prostatitis is a condition that significantly hampers the quality of life (QoL) of the majority of patients affected, because it is not only a physical condition but also a psychological distress. The impact of CBP is similar to those patients who had a myocardial infarction, unstable angina or Crohn’s disease [2]. Pathophysiology of CBP at the moment remains unknown although some mechanisms of actions, like infectious, immunological, neurological, endocrine and psychological, have been postulated [3]. Commonly patients are treated with antibiotics alone as recommended by the European Urology guidelines. In particular, for chronic bacterial prostatitis, fluoroquinolones, thanks to their favourable pharmacokinetic properties, prostate penetration, bioavailability and excellent activity against typical/atypical pathogens together with a good safety profile, are considered drugs of choice. The use of fluoroquinolones, a broad-spectrum antibiotic, is strengthened by the well-known evidence that CBP is commonly caused by Gram-negative bacteria such as Escherichia coli, and also by the recent consideration of some authors that reported an emerging prevalence of Gram-positive and atypical bacteria together with anaerobes [4,5]. Usually high-doses and a long-lasting therapy are preferred because only low-molecular-weight and lipid-soluble drugs, not tightly connected to plasma proteins, are able to penetrate the epithelial membrane, even considering reported side-effects (gastrointestinal disorders and development of antibiotics resistance) [6-8]. This approach, although recommended, may not be enough, considering patients’ and urologists’ high rate of dissatisfaction. Furthermore, given the overlapping of lower urinary tract symptoms, antibiotics alone could be inadequate. The main purpose of the therapy is to eradicate uropathogens without forgetting to relieve symptoms that mainly act on QoL. Considering the heterogeneous nature of chronic prostatitis and recurrence rate of this disorder, a multimodal approach to the prolonged antibiotic therapy may be helpful for the solution of the problem at hand. The use of plant-derived products is reaching popularity in North America and Europe and is often one of the treatments of choice for chronic conditions [9]. Main advantages of such a therapy are unique mechanisms of action, response against LUTS, low side-effects profile, low cost and high level of acceptance by patients. Main disadvantages are unknown drug interactions and meaningless labels [9]. Serenoa Repens, saw palmetto extract, is the most commonly used phyto-therapy in urology and has an action against type I-II 5-α-reductase (conversion of testosterone in dihydrotestosterone), anti-inflammatory effect by inhibition of arachidonic acid metabolites and also an anti-edematous effect [10]. Arbutin, the active principle of bearberry, is an antioxidant agent with an anti-inflammatory action through lypopolisaccharide induced production of NO and expression of iNOS and COX-2 [11]. The use of probiotics, in particular lactobacillus sporogens, is a good alternative for the treatment and prevention of urinary tract infection (UTI) through different mechanisms including attachment to the uroepithelial cells and direct antimicrobial activity [12]. Lactobacillus is an important part of the normal flora, which is commonly found in the mouth cavity, gastrointestinal tract and genitourinary tract. Reduction in number of lactobacillus increases the risk of UTI [13].

In the article we report results of an association therapy with third generation fluoroquinolone (prulifloxacin 600 mg) and nutraceutical supplement (Serenoa repens 320 mg, Lactobacillus Sporogens 200 mg, Arbutin 100 mg) in the treatment of chronic bacterial prostatitis.

## Methods

From January 2012 to December 2012 a total of 210 patients with an average age of 36.6 (19–54) were enrolled in the study. In particular all of the patients were affected by chronic bacterial prostatitis and in accordance with the definition of the disease were positive to the Meares-Stamey test and symptoms duration was > 3 months (dysuria, pelvic pain and/or discomfort). The Meares-Stamey test, also known as 4-glass test, is the standard method of assessing inflammation and presence of bacteria in the lower urinary tract of men presenting CBP. The test has been performed on each patient before and after the therapy. The Meares-Stamey evaluation allows the collection of four samples: first voided urine (VB1) that represents urethra, mid-stream urine (VB2) that represents bladder, expressed prostatic secretion (EPS) and post-prostatic massage urine (VB3) that represent the prostate. It is considered positive when we have urophathogen colony-forming units (CFU)/mL ≥10^3^.

The NIH-Chronic Prostatitis Symptom Index (NIH-CPSI) is a questionnaire with 13 questions developed to evaluate symptoms and quality of life in men with chronic prostatitis/chronic pelvic pain syndrome (CP/CPPS). Every question has a score differing by the answer, and the questionnaire has a total score ranging from 0 to 43. The score is divided by three subscales: pain (score range 0–21), urinary symptoms (score range 0–10) and quality of life (QoL) (score range 0–12). The pain subscale includes six items scoring from 0 to 1, one scoring from 0 to 5 and one scoring from 0 to 10. The urinary subscale consists of two items, both of them scored from 0 to 3. The QoL subscale includes further two items scored from 0 to 3, and one scored from 0 to 6. The sum of all single scores is the total score. The reason every item has a different maximum score is because they have a different potential. NIH-CPSI characteristics are: good reliability, validity, and responsiveness to change. It has been used in many large-scale trials regarding CP/CPPS as the primary outcome variable [14]. Exclusion criteria of the study were: positivity to Chlamydia trachomatis, Ureaplasma urealiticum, Mycoplasma, Neisseria gonorrhoeae, herpes simplex viruses (HSV 1/2) and human papillomavirus (HPV); age less than 18 years; history of neurological disease, urinary stones or cancer; allergy to fluoroquinolones; refusal to sign the informed consent; incomplete follow-up time. Taking into account the aforementioned criteria, 57 patients were excluded. The purpose of the study was to evaluate the efficacy of a short lasting therapy with a fluoroquinolone, recommended as first choice in this disease, in association with a nutraceutical supplement. In particular prulifloxacin, a third generation fluoroquinolone, has demonstrated to be not-inferior to levofloxacin in the treatment of urinary tract infections [15]. Treatment schedule was based on oral prulifloxacin 600 mg (Unidrox®) 1 tablet daily for 21 days and an association of Serenoa repens 320 mg, Lactobacillus Sporogens 200 mg, Arbutin 100 mg (Lactorepens®) 1 tablet daily for 30 days. All eligible patients signed an informed consent to participate in the study and everyone, before the beginning of the treatment, underwent medical history, urological examination, Meares-Stamey test, PSA evaluation and compiled the NIH-CPSI questionnaire (Italian version). In order to exclude the presence of other urological disease simulating a prostatitis, every patients had a bladder and prostate ultrasound and compiled a voiding diary. At 2 months, 4 months and at 6 months from the start of the therapy a follow-up examination was scheduled, and in particular every patient underwent the same tests as at the beginning of the treatment. Randomization was carried out using a double-blind ratio of 1:1. Patients were divided in two groups: Group A (76 patients) receiving antibiotic alone and Group B (77 patients) receiving an association of antibiotic and supplement. Outcomes of the study were: clinical resolution of the disease and relief of the symptoms. Clinical resolution was defined as being asymptomatic for a minimum of 1 month, and clinical failure as the persistence of symptoms together with clinical signs (Meares-Stamey positivity) after treatment or after the suspension of the therapy. Regarding symptoms evaluation, we used the NIH-CPSI subscale (urinary symptoms - score range 0–10) and we defined asymptomatic a patient with a score of 0. Furthermore, spontaneously reported adverse events or those noted by the investigator were recorded during the whole study period.

Statistical analysis was carried out with BMDP statistical software, version 7 (Statistical Solutions, Saugus, MA) and SPSS (Chicago, IL, version 15.00 for Windows). Statistical significance was achieved if the p-value was <0.05. All reported P-values are two-sided.

The ethical committee approval (Sapienza Rome University - Department of Gynecological-Obstetric Sciences and Urological Sciences - Ethical Committee) was obtained and all treatments applied are part of routine standard care. The study was conducted in line with European Urology and Good Clinical Practice guidelines, with ethical principles laid down in the latest version of the Declaration of Helsinki. Every patient signed an informed consent to participate in the study.

## Results

**Figure 1:**
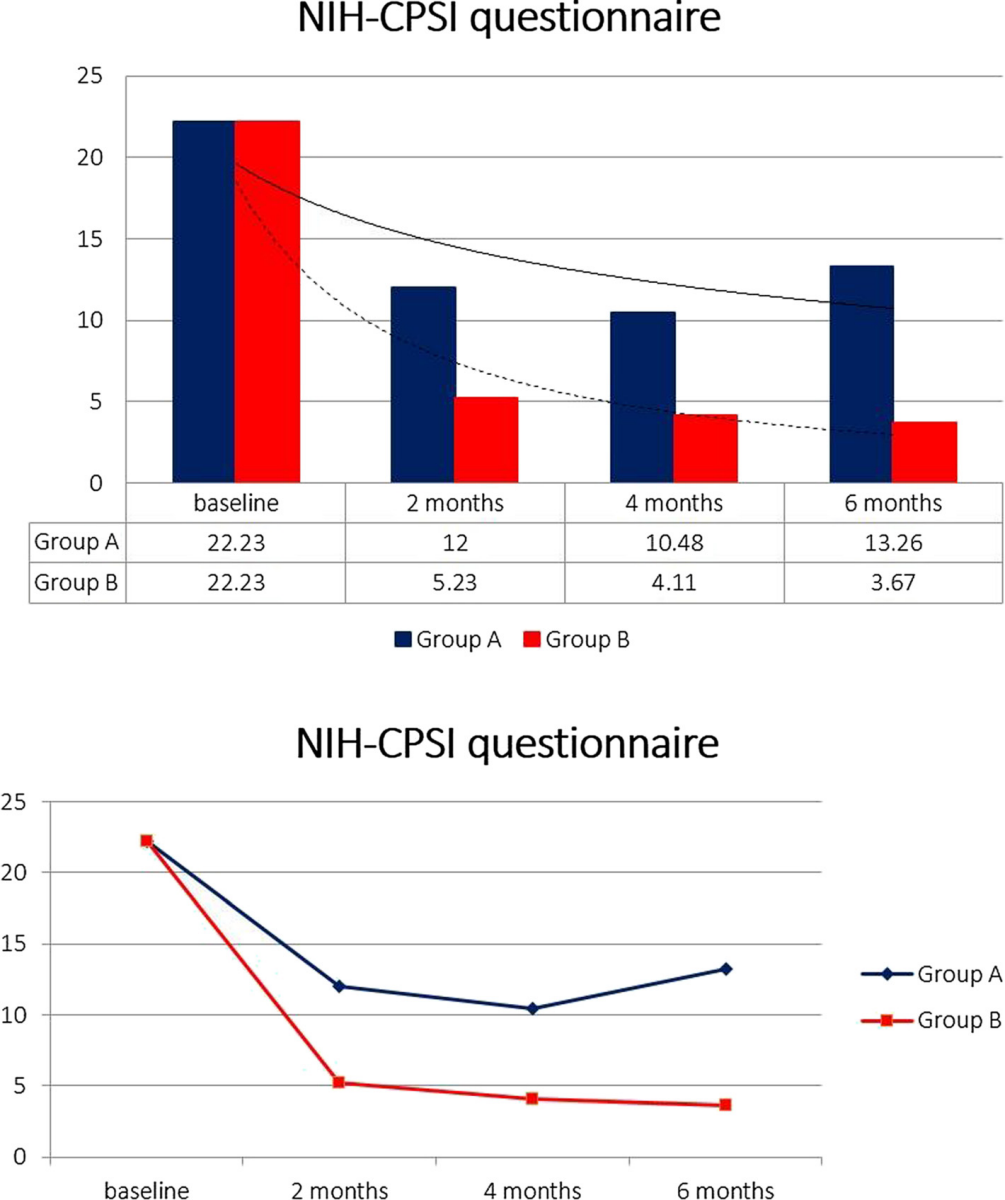
**NIH-CPSI questionnaire.** NIH-CPSI questionnaire score at baseline, 2nd, 4th and 6th month.

**Figure 2:**
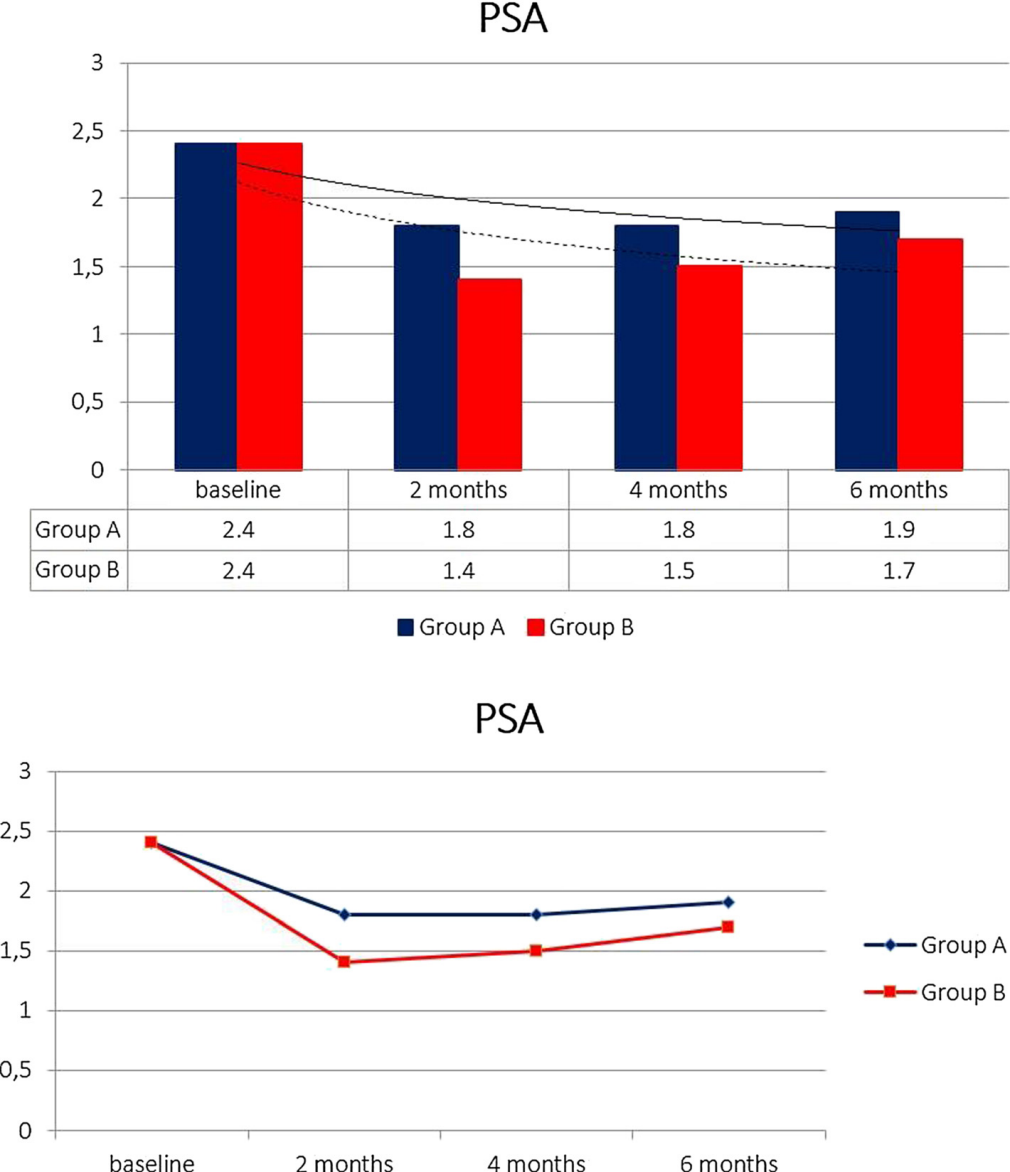
**PSA.** Serum PSA at baseline, 2nd, 4th and 6th month.

**Figure 3:**
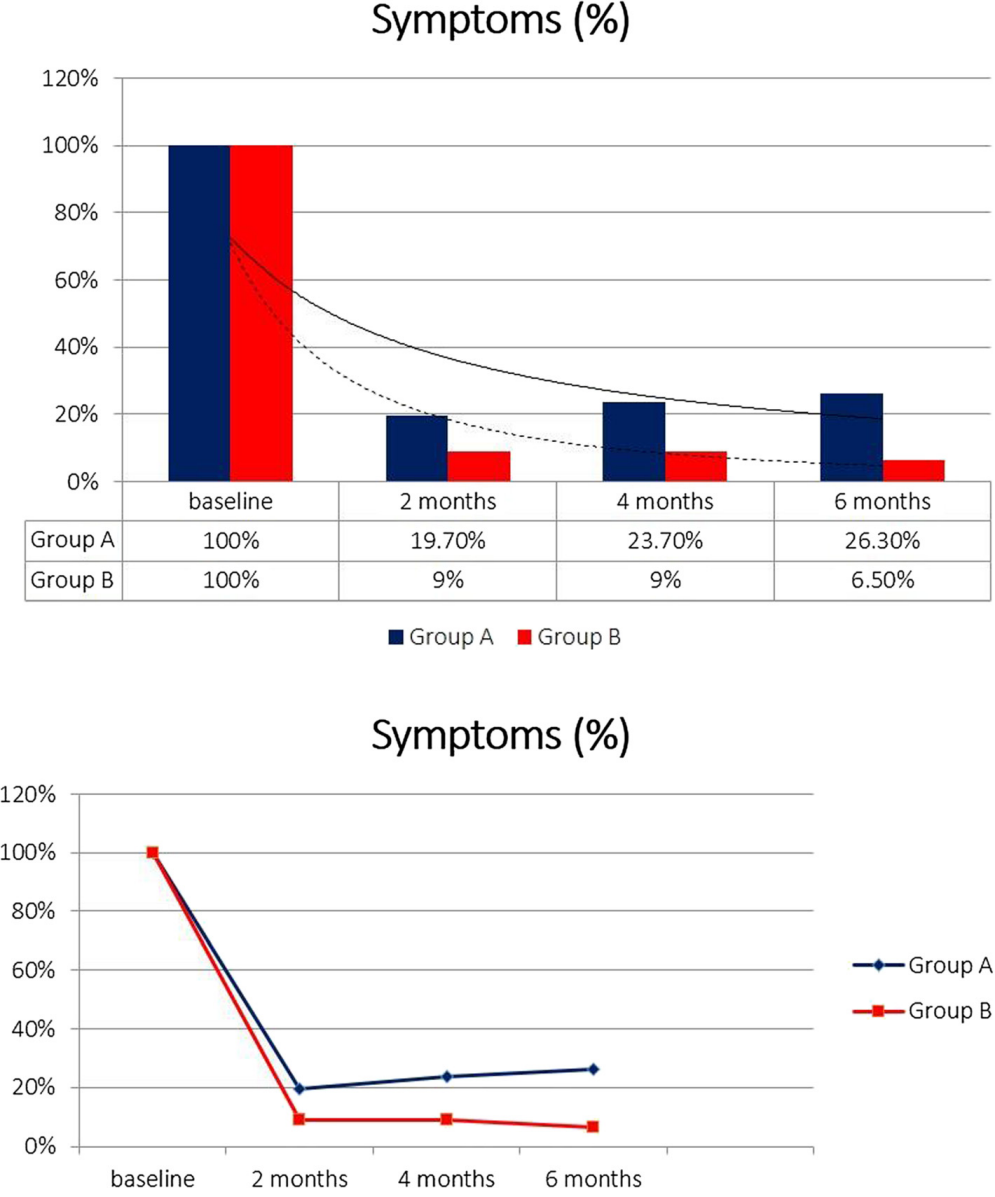
**Symptoms.** Presence of symptoms at baseline, 2nd, 4th and 6th month.

Including an initial population of 210 patients and considering the exclusion criteria, 57 patients have been excluded. In particular, 21 patients were positive to Chlamydia trachomatis/Ureaplasma urealiticum, 10 patients positive to Mycoplasma and 8 positive to HPV (2 patients Chlamydia together with Mycoplasma). Furthermore, 7 patients were positive for urinary stones and only one referred an allergy to fluoroquinolones. The follow-up time of 6 months was not reached by 10 patients. Patients’ characteristics are listed in Table 1. In our study, in total, 153 patients have been enrolled. Mean age of patients was 36.6 ± 6.4 and median symptom time was 16.4 months (4–24). Considering uropathogens found in the Meares-Stamey test, there was a balance between Gram-positive and Gram-negative bacteria: 73 (47.7%) Gram + and 80 (52.3%) Gram – (Table 2). Biological recurrence at 2 months (positivity of Meares-Stamey test) in Group A was observed in 21 patients (27.6%) and in Group B in 6 patients (7.8%). Uropathogens found at the first follow-up were for the majority Gram – (E. coli and Enterobacter spp.) and are listed in Table 2. Patients with recurrence, in accordance with antibiogram, received an alternative antibiotic therapy without uptake of any supplement. Looking at the NIH-CPSI questionnaire before the therapy (baseline) we reported a score of 22.23 ± 5.55. All patients compiled the questionnaire after 2 months, 4 months and 6 months from the beginning of the therapy and results are as follows: Group A 12.00 ± 4.34, Group B 5.23 ± 2.76 at 2 months; Group A 10.48 ± 4.14, Group B 4.11 ± 2.32 at 4 months; Group A 13.26 ± 4.88, Group B 3.67 ± 1.98 at 6 months (Figure 1). Data regarding the NIH-CPSI questionnaire divided in the 3 subscales are listed in Table 3. Furthermore, the difference between the Groups is always statistically significant with a p value at 2, 4 and 6 months always < 0.001. Serum median PSA at baseline was 2.4 ± 1.8 ng/ml. PSA values after the therapy were: Group A 1.8 ± 1.2, Group B 1.4 ± 0.9 at 2 months; Group A 1.8 ± 1.2, Group B 1.5 ± 0.9 at 4 months; Group A 1.9 ± 1.2, Group B 1.7 ± 1.0 at 6 months (Figure 2). After the therapy the difference between the groups isn’t statistically significant with a p value at 2, 4 and 6 months always > 0.05. Symptoms related to CBP have been reported by 15 patients (19.7%) in Group A and 7 patients (9%) in Group B at 2 months, 18 patients in Group A (23.7%) and 7 patients (9%) in Group B at 4 months, 20 patients (26.3%) in Group A and 5 patients (6.5%) in Group B at 6 months (Figure 3). Symptoms reported by the patients are: burning, tenesmus, urgency, frequency, post voiding dribble and micturition with pain. During the follow-up time all the symptoms improved in both groups with a difference at 2, 4 and 6 months not always statistically significant; in particular, the difference, is significant only at 4 and 6 months with a p value respectively of 0.012 and < 0.001. All included patients received therapy properly without any dose/tablet variation and with a compliance of 100%. No side effects were reported with either therapy scheme (antibiotic vs antibiotic + compound). Only patients with a biological recurrence received an additional antibiotic course in accordance with antibiogram.

**Table 1:** Patients characteristics

	**Group A**	**Group B**
No. of patients*	76	77
Median age (±S.D.) (years)*	35.9 (6.2)	37.1 (6.5)
*Sexual bahaviour (%)*		
Only 1 partner*	50 (65.8)	52 (67.5)
2 or more partners*	26 (34.2)	25 (32.5)
*Contraceptive method (%)*		
No contraceptive**	28 (36.7)	31 (40.3)
Condom*	9 (11.8)	9 (11.7)
Coitus interruptus**	39 (51.3)	37 (48.1)
*Urinary symptoms (%)*		
Burning*	65 (85.6)	65 (84.4)
Tenesmus*	24 (31.6)	26 (33.8)
Pain**	40 (52.6)	34 (44.2)
Urgency**	40 (52.6)	32 (41.6)
Frequency**	46 (60.5)	55 (71.4)
Beginning of CBP (months)*	15.9	17.4

*not-statistically significant difference between Group A and B.

**statistically significant difference between Group A and B.

**Table 2:** Uropathogens characteristics and disease recurrences

	**Group A**	**Group B**
*Uropathogens (baseline) (%)*		
*Gram negative*	41 (53.9)	39 (50.6)
Escherichia coli	33 (80.5)	33 (84.6)
Proteus mirabilis	3 (7.3)	1 (2.6)
Klebsiella spp.	7 (17.1)	5 (12.8)
Serratta spp.	2 (4.88)	4 (10.3)
Pseudomonas aeruginosa	0 (0)	0 (0)
Enterobacter spp.	20 (48.8)	15 (38.5)
*Gram positive*	35 (46.1)	38 (49.4)
Enterococcus spp.	25 (71.4)	27 (71)
Staphylococcus saprophyticus	10 (28.6)	10 (26.3)
Staphylococcus epidermidis	2 (5.7)	4 (10.5)
Staphylococcus aureus	1 (2.8)	0 (0)
Streptococcus B group	6 (17.1)	4 (10.5)
Recurrences (2 months) (%)	21 (27.6)	6 (7.8)
*Uropathogens (2 months) (%)*		
*Gram negative*	21 (95.4)	6 (100)
Escherichia coli	17 (81)	4 (66.7)
Proteus mirabilis	0 (0)	0 (0)
Klebsiella spp.	2 (9.5)	1 (16.7)
Serratta spp.	0 (0)	0 (0)
Pseudomonas aeruginosa	0 (0)	0 (0)
Enterobacter spp.	2 (9.5)	1 (16.7)
*Gram positive*	1 (4.5)	0 (0)
Enterococcus spp.	1 (100)	0 (0)
Staphylococcus saprophyticus	0 (0)	0 (0)
Staphylococcus epidermidis	0 (0)	0 (0)
Staphylococcus aureus	0 (0)	0 (0)
Streptococcus B group	0 (0)	0 (0)

**Table 3:** NIH-CPSI questionnaire results

	**Group A**	**Group B**
*Symptoms score (NIH-CPSI questionnaire 0–43) (±S.D.)*		
Baseline	21.85 ± 5.23	22.56 ± 5.78
2 months	12.00 ± 4.34	5.23 ± 2.76
4 months	10.48 ± 4.14	4.11 ± 2.32
6 months	13.26 ± 4.88	3.67 ± 1.98
*Pain score (NIH-CPSI questionnaire 0–21) (±S.D.)*		
Baseline	11.65 ± 3.23	12.00 ± 2.78
2 months	6.20 ± 2.35	2.13 ± 1.90
4 months	5.82 ± 2.10	1.65 ± 1.17
6 months	8.06 ± 2.81	1.25 ± 0.99
*Urinary score (NIH-CPSI questionnaire 0–10) (±S.D.)*		
Baseline	4.65 ± 2.11	4.26 ± 2.28
2 months	2.35 ± 1.35	1.34 ± 0.89
4 months	2.10 ± 1.15	1.11 ± 0.89
6 months	2.65 ± 1.44	1.01 ± 0.76
*Symptoms (%)*		
Baseline	76 (100)	77 (100)
2 months	15 (19.7)	7 (9)
4 months	18 (23.7)	7 (9)
6 months	20 (26.3)	5 (6.5)
*QoL (NIH-CPSI questionnaire 0–12) (±S.D.)*		
Baseline	5.76 ± 2.44	5.66 ± 2.39
2 months	2.89 ± 1.68	1.85 ± 1.00
4 months	2.60 ± 1.43	1.66 ± 0.95
6 months	3.09 ± 1.74	1.40 ± 0.94

## Discussion

Chronic bacterial prostatitis (CBP) type II, although its prevalence is low, is a frustrating condition for patients because it is characterized by a high impact on quality of life (QoL) and also by a frequent recurrence rate [16,17]. This condition represents a challenge for urologists and, to date, the optimal management remains controversial and the only recommended treatment is antibiotic therapy and surgery in those limited cases with severe complications [18,19]. Other treatments, commonly adopted, are anti-inflammatory drugs, α-blockers, phytotherapy and alternative therapies such as biofeedback, psychotherapy and acupuncture [20]. Long-lasting therapy with fluoriquinolones antibiotics is one of the best treatments in patients with CBP because of a pharmacokinetic favourable profile and in particular ciprofloxacin and levofloxacin are recommended. The latter also has a favourable action against Gram + pathogens. Prulifloxacin, a third generation fluoroquinolone, has been approved for the treatment of lower urinary tract infection. Furthermore, it has demonstrated its ability to better penetrate into prostatic tissue compared to other quinolones, thus confirming a potential therapeutic role in the treatment of bacterial prostatic infections [21]. Giannarini et al., comparing a 4-weeks course of prulifoxacin in comparison with levofloxacin in the treatment of chronic bacterial prostatitis, report, at 6 months, a microbiological eradication of 72.73% and 71.11%, repectively, and a reduction in the NIH-CPSI of 10.75 and 10.73. The authors conclude that prulifloxacin is at last as effective and safe as levofloxacin [15]. Serenoa repens, saw palmetto extract, the most widely used supplement for lower urinary tracts (LUTS), and in particular for prostatic infection and inflammation, has a multimodal effect that is explained with a set of different mechanisms of action: selective antagonism of the link between dihydrotestosterone and androgen receptor; inhibition of 5-alpha-reductase, involved in the transformation of testosterone to its biologically active metabolite, that stimulates cell proliferation and hypertrophy of the prostate tissue; anti-inflammatory and anti-oedema effect, demonstrated by reduced capillary permeability induced by histamine; anti-estrogenic effect given by decline in estrogen receptors, which seems to potentiate the action of hormones in the development of BHP [22,23]. Unlike 5-α-reductase inhibitors (5-ARI), a selective competitive inhibitors of α-reductase type II that is more specific for prostate, serenoa repens isn’t selective and acts against both types (type I and II). Cai et al., comparing the usage of prulifloxacin alone with plurifloxacin in association with serenoa repens, urtica dioica, quercitin and curcumin in the treatment of chronic bacterial prostatitis type II, report a statistically significant difference in QoL and symptoms (absence of symptoms: 27% vs 89.6%) [17]. Probiotics are important to reduce gastrointestinal side-effects caused by the prolonged use of broad-band antibiotics. Considering that pathogens found in the prostate often derive from intestinal bacterial overgrowth, it is important to remark the role of probiotics in maintaining a regular intestinal bacterial flora [24]. Some authors have postulated that urethral dysbacteriosis is one of the primary causes of CP, further contributing to its recidivity and refractoriness [12]. In particular, when this condition occurs, the urethral microflora infects the prostate through prostatic reflux of urine into prostatic ducts causing bacterial prostatitis and inflammation [25]. Overused and overprescribed antibiotics are usually the cause of urethral dysbacteriosis while probiotics are considered a viable potential alternative for treating and preventing prostatitis and all urinary tract infections. Probiotics, with different mechanisms of action, have the ability to attach to uroepithelial cells and obtain direct antimicrobial activity [26]. The reason we suggest using probiotics in association with a long-course antibiotic is to rebalance intestinal bacterial flora and to avoid any urinary tract dysbacteriosis and to prevent UTI recurrences [12]. One of the limits of many studies on chronic prostatitis is that they focus only on infection eradication instead of symptoms improvement. Inflammation, together with neuromuscular spasm, is often the most important cause of chronic prostatitis symptoms [27]. Arbutin, a glycoside extracted from bearberry plant, is a traditional supplement for treating UTI mainly because of its anti-inflammatory effect due to antioxidant capability dispatched on lypopolisaccharide, induced production of NO and expression of iNOS and COX-2 [11].

Our study has been drawn to evaluate the efficacy of a short-course, broad band antibiotic therapy (21 days of prulifloxacin 600 mg) to eradicate pathogens in patients with CBP and to evaluate if an association with a supplement (30 days of serenoa repens 320 mg, lactobacillus sporogens 200 mg and arbutin 100 mg) is able to prevent recurrences and improve symptoms in those patients with this particularly frustrating condition. To evaluate the outcomes, patients, divided in two different groups (A and B), have been submitted to different therapies: antibiotic or antibiotic plus supplement. Looking at the results we can notice, with a statistically significant difference, that patients treated with the association of compounds obtain better effects as demonstrated with the NIH-CPSI questionnaire. Analysing first, second and third follow-up, patients in Group A obtain an improvement at 2 and 4 months while there is a decrease in results at the 6th month. Patients in Group B obtain better results in comparison with Group A, and furthermore the NIH-CPSI score continues to also decrease at the 6th month follow-up. Symptoms, probably the most invalidating condition for the patients, have been reported by patients in Group A to improve only at 2nd month follow-up while at 4th and 6th month there is a turnaround. Patients in Group B continued to report benefits till the end of follow-up time. Reported symptoms together with the NIH-CPSI questionnaire score are better in all patients treated with antibiotic plus supplement, confirming more stable and long-standing results in QoL that is always the main target of CBP patients. Prostatic specific antigen (PSA) decreases in both groups with a lower value in Group B probably because of the effect of serenoa repens that acts on type I-II 5-α-reductase, inhibiting testosterone conversion to its active metabolite. With regards to this, it is important to remark that the prostate specific antigen does not always increase during a prostatitis and does not seem to be systematically correlated to prostate inflammation [28]; this is the most important reason why it isn’t a good parameter to show a therapy’s response. Finally we found a difference between Group A and B in uropathogens eradication; in particular we have a recurrence rate at 2 months of 27.6% and 7.8%, respectively. Pathogens found during follow-up are often different from pathogens found at baseline. We think that the main reason is because repeated cycles of antibiotic therapy are never able to prevent bacteria relapsing [29] and this is why we can improve results by adding compounds to antibiotic therapy alone. In particular, results, show that association therapy obtains better results in avoiding the majority of recurrences and prolonging the recurrence-free time.

Limitations of the study are: small number of patients, not randomized double-blind placebo controlled trial, impossibility to attribute to single components of the compound the action, antibiotic therapy duration scientifically debated, and difficulty to evaluate if the results in the patients with recurrence have been altered by different antibiotic administered in accordance with antibiogram.

## Conclusions

In summary, a broad band, short-lasting antibiotic therapy in association with a nutritional supplement (serenoa repens, lactobacillus sporogens and arbutin) show better control and recurrence rates on patients affected by chronic bacterial prostatitis, in comparison with compared to antibiotic treatment alone.

## Competing interests

The authors declare that they have no competing interests.

## Authors’ contributions

GMB analysed data and drafted the manuscript. RG acquired data. MF participated in interpretation of data. ST and FDG gave their contribution in acquiring data. ODC and VG critically revised the manuscript. EDB participated in the conception and design of the study. All authors read and approved the final manuscript.

## Pre-publication history

The pre-publication history for this paper can be accessed here:

http://www.biomedcentral.com/1471-2490/14/53/prepub
